# Age-dependent associations of peripheral inflammatory biomarkers with cognition and brain structural abnormalities in patients with Alzheimer’s disease and Alzheimer’s disease-related mild cognitive impairment

**DOI:** 10.3389/fneur.2026.1868357

**Published:** 2026-09-08

**Authors:** Yida Wang, Min Cheng, Zunjing Liu

**Affiliations:** Department of Neurology, Peking University People's Hospital, Beijing, China

**Keywords:** Alzheimer’s disease, brain atrophy, chronic peripheral inflammation, cognition, inflammatory biomarkers

## Abstract

**Background:**

Alzheimer’s disease (AD) is one of the most prevalent neurodegenerative conditions. Chronic peripheral inflammation has been implicated in the pathophysiology of AD. Given their accessibility and cost-effectiveness, peripheral inflammatory biomarkers hold promise as potential indicators for AD. However, their associations with cognitive function and brain structural abnormalities may vary across patients and age groups.

**Methods:**

Patients diagnosed with AD or AD-related mild cognitive impairment (AD-MCI) were included. Peripheral inflammatory biomarkers, including white blood cell count (WBC), neutrophil count (NEU), lymphocyte count (LYM), and monocyte count (MONO), were obtained from complete blood count (CBC). Composite inflammatory biomarkers were calculated from the aforementioned parameters and included the neutrophil-to-lymphocyte ratio (NLR), monocyte-to-lymphocyte ratio (MLR), neutrophil-to-platelet ratio (NPR), neutrophil-monocyte-to-lymphocyte ratio (NMLR), systemic immune-inflammation index (SII), systemic inflammation response index (SIRI), and aggregate index of systemic inflammation (AISI). We examined the associations of peripheral blood inflammatory biomarkers and calculated composite inflammatory biomarkers with cognitive function, global cortical atrophy, and hippocampal atrophy. Interaction and stratified analyses were employed to investigate the modifying effects of age and sex on these associations.

**Results:**

The study included a total of 395 patients. Within the overall sample, almost all inflammatory biomarkers did not exhibit significant associations with cognitive function, global cortical atrophy, or hippocampal atrophy. Interaction analyses revealed significant interactions between age and several inflammatory biomarkers, while no significant interactions were detected concerning sex. Stratified analyses indicated that among patients younger than 75 years, several composite inflammatory biomarkers were significantly associated with cognitive function, global cortical atrophy, and hippocampal atrophy, whereas no significant associations were observed in patients aged 75 years or older.

**Conclusion:**

In patients with AD and AD-MCI, the associations between peripheral inflammatory biomarkers and both cognitive impairment and structural brain abnormalities were found to be age-dependent, with stronger associations observed in patients younger than 75 years. Several composite inflammatory biomarkers showed more consistent associations and may help characterize heterogeneity within this clinical population, although their prognostic and diagnostic value requires further validation.

## Highlights

- Peripheral inflammatory biomarkers were evaluated in AD and AD-MCI.
- Most biomarkers were not significant in the overall sample.
- Age modified these biomarker-outcome associations.
- Several composite biomarkers were associated with cognition and brain atrophy in patients aged < 75 years.
- No significant associations were found in patients aged 75 years or older.

## 1 Introduction

AD represents the most prevalent form of dementia among the elderly population and constitutes a primary contributor to the deterioration of daily living activities and the impairment of social functioning (1). As the global population continues to age at an accelerated pace, the burden of AD has been progressively escalating, thereby posing a significant public health challenge (2–4). Pathological changes associated with AD may persist for several years prior to the manifestation of clinical symptoms (5), and by the time characteristic dementia symptoms emerge, the optimal period for intervention may have already elapsed. Consequently, the early identification of risk factors is critically important. Although biomarkers such as amyloid-β (Aβ) positron emission tomography (PET) imaging and the quantification of Aβ and tau proteins in cerebrospinal fluid (CSF) can aid in early diagnosis (6), these methods are invasive and costly, which limits their utility as screening tools. Therefore, there is a pressing need to investigate alternative markers that are more accessible and easier to obtain.

Currently, the involvement of neuroinflammation, mediated by microglia and astrocytes, in the initiation and progression of AD is well-established (7–10). At the same time, accumulating evidence suggests that chronic peripheral inflammation may also contribute importantly to the pathogenesis of AD (11–13). The status of peripheral inflammation may affect the neurodegenerative process through mechanisms such as alterations in blood–brain barrier (BBB) function (14–16) and the transmission of peripheral immune signals to the central nervous system via the vagus nerve (17, 18). This suggests an important connection between systemic immune imbalance and pathological changes in the brain.

Inflammatory biomarkers can be classified into peripheral inflammatory biomarkers and calculated composite inflammatory indices. Composite indices integrate the relative changes among blood cell populations and may provide a broader representation of systemic inflammatory status than any single cell count. The NLR reflects the balance between neutrophil-associated innate inflammatory activity and lymphocyte-related adaptive immune regulation (19), whereas the MLR reflects monocyte-associated inflammation relative to lymphocyte status (20). The NMLR integrates neutrophil- and monocyte-associated innate inflammatory activity relative to lymphocytes (21). The SII combines neutrophil, lymphocyte, and platelet counts (22, 23), while the SIRI integrates neutrophil, monocyte, and lymphocyte counts. The AISI incorporates neutrophils, monocytes, platelets, and lymphocytes and may provide a more comprehensive summary of systemic inflammatory imbalance (24). These composite indices have been investigated as accessible markers of peripheral inflammation; however, they remain nonspecific and indirect measures and do not directly assess immune or neuroimmune function.

However, previous studies examining the relationships between chronic peripheral inflammation, cognitive function, and brain structural abnormalities in AD have produced inconsistent results (25–27), underscoring the complexity and heterogeneity of chronic peripheral inflammation under varying conditions. Age is a significant determinant of inflammatory status. Aging is accompanied by immune remodeling and a persistent low-grade inflammatory state, commonly termed “inflammaging” (28). This complex process cannot be adequately characterized simply as either a decline or an improvement in immune function. Furthermore, peripheral inflammatory status may vary according to sex. During the perimenopausal period, declining estrogen levels are linked to heightened systemic inflammation, potentially exacerbating central nervous system inflammation and elevating the risk of AD in women (29, 30). Consequently, identifying the characteristics of chronic peripheral inflammation across different populations is crucial for enhancing our understanding of the underlying inflammatory mechanisms.

This study systematically assessed the associations between inflammatory biomarkers and cognitive impairment, as well as brain structural abnormalities, within a clinical population of patients with AD and AD-MCI. We further investigated whether age and sex modulate the relationships between inflammation and cognitive impairment, as well as brain structural changes, with the aim of characterizing heterogeneity in the inflammatory correlates of cognitive and structural brain abnormalities within the patient population.

## 2 Methods

### 2.1 Study population

This study was reviewed and approved by the Ethics Committee of Peking University People’s Hospital (2025PHB335-001) and was conducted in accordance with the Declaration of Helsinki. The study used existing clinical and laboratory data obtained as part of routine medical care, and no additional intervention or research-specific blood sampling was performed.

Patients aged 60 years or older who attended the Department of Neurology at Peking University People’s Hospital between January 2024 and December 2025 and were diagnosed with AD or AD-MCI were enrolled in a consecutive manner. Diagnoses were established in accordance with the 2011 diagnostic guidelines provided by the National Institute on Aging and the Alzheimer’s Association (NIA-AA) (31, 32). Patients meeting the diagnostic criteria for mild cognitive impairment due to Alzheimer’s disease were classified as having AD-MCI, whereas those meeting the criteria for dementia due to Alzheimer’s disease were classified as having AD.

Exclusion criteria comprised the following: the presence of acute inflammatory diseases within 1 month prior to the visit, such as acute infection, severe trauma or surgery, acute myocardial infarction, or acute cerebral infarction; hematological disorders including leukemia or lymphoma; the use of glucocorticoids, immunosuppressants, or other biologic agents impacting the immune system within 1 month prior to the visit; medical history, physical signs, or auxiliary examination findings suggesting that cognitive decline could be attributed to alternative conditions, such as vascular dementia, frontotemporal dementia, encephalitis, or encephalopathy; and incomplete laboratory test results or failure to complete the standardized Mini-Mental State Examination (MMSE) during the visit.

This observational cross-sectional study consecutively included all eligible patients with AD or AD-MCI who attended our center during the predefined study period. Because previous studies reported heterogeneous associations between peripheral inflammatory biomarkers and cognitive or structural brain outcomes, a reliable anticipated effect size was not available. Of the 537 potentially eligible patients screened, 395 met the eligibility criteria and were included in the final analysis.

### 2.2 Data collection

Demographic and clinical data were collected, encompassing age, sex, and educational attainment categorized as primary school or below, middle school, college or junior college, and master’s degree or above. Marital status was classified as married or other. Anthropometric measurements included height and weight. Medical history was documented for conditions such as hypertension, diabetes mellitus, coronary atherosclerotic heart disease, and stroke. Additionally, histories of smoking and alcohol consumption were recorded.

CBC results obtained as part of standard clinical care during the patients’ initial visit were extracted from their clinical records, including measurements of white blood cell count (WBC), neutrophil count (NEU), lymphocyte count (LYM), and platelet count (PLT). Composite inflammatory biomarkers were calculated using specified formulas:

1. Systemic immune inflammation index (SII) = PLT × NEU/LYM;
2. Systemic inflammation response index (SIRI) = MONO × NEU/LYM;
3. Neutrophil-to-lymphocyte ratio (NLR) = NEU/LYM;
4. Monocyte-to-lymphocyte ratio (MLR) = MONO/LYM;
5. Neutrophil-to-platelet ratio (NPR) = NEU/PLT;
6. Neutrophil-monocyte-to-lymphocyte ratio (NMLR) = (NEU + MONO)/LYM;
7. Aggregate index of systemic inflammation (AISI) = NEU × MONO × PLT/LYM.

Cognitive function was evaluated utilizing MMSE, a 30-point screening instrument for global cognitive performance, with lower scores generally indicating greater cognitive impairment. All MMSE evaluations were administered in person by trained neurological assessors employing a standardized protocol. To ensure consistency and comparability of data, only the MMSE score obtained during the patient’s initial visit was recorded as the measure of cognitive function.

Structural abnormalities of the brain were assessed through neuroimaging data. Global cortical atrophy was evaluated using the Global Cortical Atrophy (GCA) scale, as described by Pasquier (33), which involves visual rating of sulcal widening and gyral thinning on axial MRI scans, with scores ranging from 0 to 3. Scores of 0–1 were classified as indicative of physiological atrophy, whereas scores of 2–3 were indicative of pathological atrophy. Hippocampal atrophy was assessed using the Medial Temporal Lobe Atrophy (MTA) scale, following the Scheltens visual rating method on coronal T1-weighted images (34), by examining hippocampal height, widening of the choroid fissure, and enlargement of the temporal horn, with scores ranging from 0 to 4. For patients aged < 75 years, MTA score ≥ 2 was defined as hippocampal atrophy, whereas for those aged ≥ 75 years, MTA score ≥ 3 was defined as hippocampal atrophy.

### 2.3 Statistics

Descriptive statistical analyses were conducted to assess baseline characteristics. Initially, the normality of continuous variables was evaluated using the Shapiro–Wilk test. Data that followed a normal distribution were presented as mean ± standard deviation (mean ± SD), and intergroup comparisons were made using the independent-samples t-test. For data that did not follow a normal distribution, values were expressed as median (interquartile range), and intergroup comparisons were performed using the Mann–Whitney *U*-test. Categorical variables were reported as frequency (percentage), with intergroup comparisons conducted using either the chi-square test or Fisher’s exact test.

To examine the associations between inflammatory biomarkers and cognitive function scores, multivariable linear regression models were employed. Each inflammatory biomarker was evaluated separately in an independent regression model, and multiple inflammatory biomarkers were not simultaneously included in the same model. Given the exploratory and hypothesis-generating nature of the study and the substantial biological correlation among several composite inflammatory indices, no formal adjustment for multiple comparisons was applied. Stepwise adjustment models were developed to account for potential confounding factors: Model 1 (unadjusted) included only the inflammatory biomarkers of interest; Model 2 (partially adjusted) incorporated adjustments for age, sex, educational level, and marital status based on Model 1; and Model 3 (fully adjusted) further included adjustments for smoking history, alcohol consumption history, and previous medical history of hypertension, diabetes mellitus, CHD, and stroke based on Model 2. To account for the potential influence of measurement scales on the β coefficients derived from multivariable linear regression models, adjusted Z-scores were computed for each inflammatory biomarker:

$$Z=\frac{X-\bar{X}}{\mathrm{SD}}$$

where *X* denotes the inflammatory biomarker,^−^*X* is the mean value, and SD is the standard deviation. By incorporating these *Z*-scores into the model, the β coefficients obtained represent the change in the dependent variable associated with a one-standard-deviation increase in the independent variable.

Additionally, logistic regression models were employed to assess the relationships between inflammatory biomarkers and structural brain abnormalities, such as global cortical atrophy and hippocampal atrophy. Covariates were adjusted similarly to the approach used in the aforementioned multivariable linear regression models, and the inflammatory biomarkers were standardized using the same adjusted *Z*-score formula.

Interaction terms were incorporated into the fully adjusted models to assess whether age and sex moderated the relationships between inflammatory biomarkers and both cognitive function and brain structural abnormalities. To more clearly demonstrate whether the associations were influenced by age, the combined cohort of patients with AD and AD-MCI was divided into two groups according to age: <75 years and ≥75 years. Fully adjusted regression models were then constructed separately for each age group.

All statistical analyses were conducted using R version 4.5.2 (R Core Team, Vienna, Austria), with a two-sided *p*-value of less than 0.05 considered indicative of statistical significance.

## 3 Results

### 3.1 Baseline characteristics

**Figure 1:**
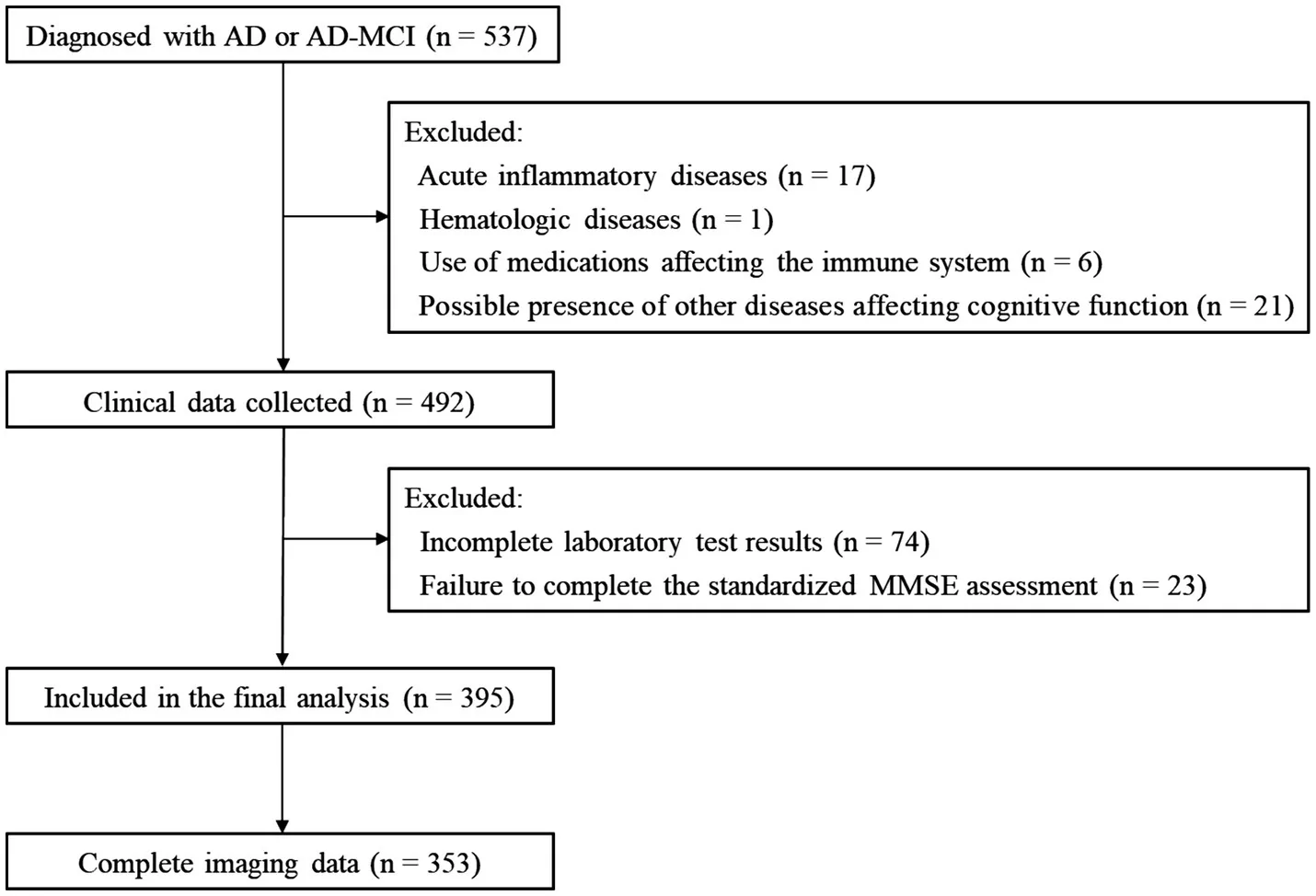
Flowchart of study participant enrollment.

A total of 537 patients who attended our hospital between January 2024 and December 2025 and were diagnosed with AD or AD-MCI were initially identified. After application of the predefined inclusion and exclusion criteria, 395 patients were included in the final analysis (Figure 1), comprising 135 patients with AD-MCI (34.2%) and 260 patients with AD (65.8%). The baseline characteristics of the overall study population are summarized in Table 1. The median age was 76 years, and 226 participants (57.1%) were female. Among participants with available education data, 60.3% had a middle-school education. Hypertension was present in 46.1% of participants, diabetes mellitus in 32.2%, coronary heart disease in 11.1%, and stroke in 7.6%. The median MMSE score was 17, reflecting the overall cognitive status of the study population. Detailed univariate comparisons of baseline characteristics between the AD-MCI and AD groups are provided in Supplementary Table S1.

**Table 1:** Baseline characteristics of the participants.

Baseline characteristics	Overall (*n* = 395)
Age (years)	76 (70, 83)
Sex	
Female	226 (57.1)
Male	169 (42.9)
Education^†^	
Primary school	56 (15.2)
Middle school	222 (60.3)
College or above	90 (24.5)
Marital status^†^	
Married	310 (81.8)
Other	69 (18.2)
Smoking	41 (10.4)
Alcohol	34 (8.7)
Hypertension	182 (46.1)
Diabetes mellitus	127 (32.2)
CHD	44 (11.1)
Stroke	30 (7.6)
BMI (kg/m^2^)	23.46 ± 3.22
WBC (10^9^/L)	5.79 (4.80, 7.11)
LYM (10^9^/L)	1.75 ± 0.63
NEU (10^9^/L)	3.66 ± 1.36
PLT (10^9^/L)	199.81 ± 55.93
SII	397 (272, 587)
SIRI	0.79 (0.51, 1.21)
NLR	2.05 (1.47, 2.81)
MLR	0.23 (0.18, 0.31)
NPR	0.02 (0.01, 0.02)
NMLR	2.31 (1.66, 3.11)
AISI	155 (96, 256)
MMSE	17 (12, 21)

^†^There were 27 missing values for educational level and 16 missing values for marital status, while no missing values were observed for the other variables.

### 3.2 Associations of inflammatory biomarkers with cognitive function and brain structural abnormalities

The associations between inflammatory biomarkers and cognitive function were assessed. In Models 1 and 2, which accounted for demographic variables, no significant linear associations were identified between any inflammatory biomarker and cognitive function scores (Supplementary Table S2). In fully adjusted model (Model 3), only SII demonstrated a statistically significant association (*β* = −0.460, *p* = 0.045), while the majority of inflammatory biomarkers continued to exhibit no linear association with cognitive function scores (Table 2). Overall, the analysis revealed no significant linear associations between most inflammatory biomarkers and cognitive function across the entire sample.

**Table 2:** Associations of inflammatory biomarkers with cognitive score, global cortical atrophy, and hippocampal atrophy.

Inflammatory biomarkers	Cognitive score		Global cortical atrophy		Hippocampal atrophy	
	*β*	*p*	OR	*p*	OR	*p*
WBC	−0.459	0.338	0.903	0.562	1.074	0.684
NEU	−0.655	0.440	0.878	0.452	1.028	0.876
LYM	0.343	0.394	0.933	0.697	0.999	0.995
PLT	−0.251	0.840	1.025	0.892	1.057	0.790
SII	−0.460	0.045*	1.087	0.651	1.146	0.491
SIRI	−0.456	0.227	1.268	0.418	1.231	0.291
NLR	−0.473	0.385	0.980	0.907	1.019	0.914
MLR	−0.297	0.207	1.701	0.057	1.188	0.338
NPR	−0.449	0.846	0.779	0.135	0.905	0.549
NMLR	−0.472	0.179	1.010	0.956	1.037	0.836
AISI	−0.541	0.414	1.353	0.300	1.310	0.246

*p < 0.05.

Subsequently, the associations between inflammatory biomarkers and brain structural abnormalities were examined separately (Table 2). In the analysis of global cortical atrophy, Model 1 indicated that the SIRI, MLR, and AISI were significantly associated with global cortical atrophy. However, after adjusting for covariates, only the MLR remained significantly associated with global cortical atrophy in Model 2 (OR = 1.607, *p* = 0.032) (Supplementary Table S3). In Model 3, none of the inflammatory biomarkers showed a statistically significant association with global cortical atrophy, although MLR showed borderline significance (OR = 1.701, *p* = 0.057). The other inflammatory biomarkers were not significantly associated with global cortical atrophy in any of the models. In the analysis of hippocampal atrophy, none of the inflammatory biomarkers showed a statistically significant association with hippocampal atrophy in any of the three models (Supplementary Table S4).

### 3.3 Modifying effects of age and sex on the associations between inflammatory biomarkers and cognitive function

To assess the potential moderating effects of variables such as age and sex on the relationships between inflammatory biomarkers and cognitive function scores, interaction models were independently developed for each biomarker in conjunction with age and sex. The findings indicated that several inflammatory biomarkers, namely LYM, NLR, NPR, and NMLR, exhibited significant interactions with age (*p* < 0.05). Conversely, the interaction analyses with sex did not yield any statistically significant results (Table 3).

**Table 3:** Interaction analyses of age, sex, and inflammatory biomarkers on cognitive function.

Inflammatory biomarkers	Age		Sex	
	*β*	*p*	*β*	*p*
WBC	0.120 (−0.663, 0.902)	0.764	−0.519 (−1.299, 0.260)	0.191
NEU	0.617 (−0.310, 1.544)	0.191	−0.456 (−1.385, 0.473)	0.335
LYM	−1.885 (−3.914, −0.144)	0.049*	−1.375 (−3.490, 0.739)	0.202
PLT	−0.017 (−0.040, 0.007)	0.176	−0.003 (−0.027, 0.021)	0.792
SII	0.003 (−0.001, 0.008)	0.137	−0.002 (−0.006, 0.002)	0.376
SIRI	0.672 (−0.833, 2.177)	0.381	0.319 (−1.398, 2.036)	0.715
NLR	1.145 (0.147, 2.142)	0.025*	−0.096 (−0.997, 0.804)	0.834
MLR	0.287 (−7.850, 10.423)	0.782	6.099 (−3.835, 16.033)	0.228
NPR	205.3 (39.5, 371.2)	0.015*	1.495 (−153.2, 156.2)	0.985
NMLR	1.052 (0.109, 1.994)	0.029*	−0.025 (−0.869, 0.819)	0.953
AISI	0.002 (−0.005, 0.008)	0.632	0.001 (−0.007, 0.008)	0.884

*p < 0.05.

The interaction analysis indicated that the relationship between inflammatory biomarkers and cognitive scores may be modulated by age. Consequently, age-stratified analyses were conducted to elucidate this potential moderating effect more clearly. Utilizing the World Health Organization’s age classification, a cutoff of 75 years was employed to categorize the study population into two groups: <75 years group and ≥75 years group. The baseline characteristics, stratified by age, are presented in Supplementary Table S5. Separate multivariable linear regression models were constructed for each age group to perform the stratified analyses. In the <75 years group, significant negative associations were found between the MMSE score and NLR (*β* = −1.541, *p* = 0.049), NPR (*β* = −1.667, *p* = 0.032), and NMLR (*β* = −1.510, *p* = 0.040). Conversely, in the ≥75 years group, no statistically significant associations were identified between any inflammatory biomarker and the MMSE score (Table 4).

**Table 4:** Associations between inflammatory biomarkers and cognitive function in participants aged < 75 years and ≥75 years.

Inflammatory biomarkers	<75 years group		≥75 years group	
	*β*	*p*	*β*	*p*
WBC	−0.485(−1.782, 0.812)	0.460	−0.168(−1.18, 0.844)	0.743
NEU	−1.036(−2.106, 0.034)	0.056	−0.256(−1.239, 0.727)	0.606
LYM	0.966(−0.376, 2.308)	0.156	0.221(−0.924, 1.367)	0.702
PLT	0.105(−1.219, 1.430)	0.875	−0.75(−1.89, 0.389)	0.194
SII	−1.353(−2.914, 0.209)	0.089	−0.248(−1.145, 0.648)	0.584
SIRI	−0.908(−2.807, 0.991)	0.345	−0.378(−1.152, 0.397)	0.336
NLR	−1.541(−3.075, −0.007)	0.049*	−0.158(−1.038, 0.723)	0.724
MLR	−0.247(−1.861, 1.367)	0.762	−0.274(−1.296, 0.749)	0.597
NPR	−1.667(−3.184, −0.151)	0.032*	0.105(−0.781, 0.99)	0.815
NMLR	−1.510(−2.961, −0.059)	0.040*	−0.17(−1.055, 0.715)	0.704
AISI	−0.691(−2.382, 1.000)	0.419	−0.488(−1.32, 0.343)	0.247

*p < 0.05.

### 3.4 Modifying effects of age and sex on the associations between inflammatory biomarkers and global cortical atrophy

To assess whether variables such as age and sex influenced the relationships between inflammatory biomarkers and brain structural abnormalities, separate interaction models were developed, incorporating inflammatory biomarkers with age and sex. In examining the interaction effects between age and inflammatory biomarkers on global cortical atrophy, the interaction terms for NEU (*p* = 0.029), SIRI (*p* = 0.021), NPR (*p* = 0.018), and NMLR (*p* = 0.048) with age were found to be statistically significant, while AISI demonstrated borderline significance (*p* = 0.059). The interaction terms for WBC, LYM, PLT, SII, NLR, and MLR with age did not achieve statistical significance. Furthermore, in the analysis of interaction effects between sex and inflammatory biomarkers on global cortical atrophy, none of the interaction terms between inflammatory biomarkers and sex reached statistical significance (Table 5).

**Table 5:** Interaction analyses of age, sex, and inflammatory biomarkers on global cortical atrophy.

Inflammatory biomarkers	Age		Sex	
	OR	*p*	OR	*p*
WBC	0.645 (0.374, 1.111)	0.114	0.912 (0.532, 1.562)	0.736
NEU	0.535 (0.306, 0.937)	0.029*	0.824 (0.476, 1.424)	0.487
LYM	1.200 (0.719, 2.003)	0.485	1.051 (0.612, 1.806)	0.857
PLT	0.878 (0.516, 1.497)	0.634	1.263 (0.732, 2.181)	0.402
SII	0.646 (0.363, 1.149)	0.137	1.106 (0.608, 2.011)	0.742
SIRI	0.255 (0.080, 0.816)	0.021*	0.585 (0.206, 1.664)	0.315
NLR	0.546 (0.295, 1.012)	0.055	0.815 (0.459, 1.449)	0.486
MLR	0.468 (0.203, 1.076)	0.074	0.737 (0.320, 1.696)	0.472
NPR	0.473 (0.254, 0.879)	0.018*	0.563 (0.313, 1.013)	0.055
NMLR	0.525 (0.278, 0.993)	0.048*	0.816 (0.452, 1.472)	0.499
AISI	0.400 (0.155, 1.035)	0.059	0.866 (0.327, 2.288)	0.771

*p < 0.05.

Based on the findings from the aforementioned interaction analyses, age-stratified analyses were conducted to more clearly illustrate the modifying effect. Distinct logistic regression models were developed for two age groups: those under 75 years and those 75 years and older. In the cohort under 75 years, significant associations with global cortical atrophy were observed for SIRI (OR = 3.485, *p* = 0.036), MLR (OR = 2.701, *p* = 0.022), and AISI (OR = 2.515, *p* = 0.041), while other inflammatory biomarkers did not demonstrate significant associations. Conversely, in the cohort aged 75 years and older, none of the inflammatory biomarkers exhibited a statistically significant relationship with global cortical atrophy (Table 6).

**Table 6:** Associations between inflammatory biomarkers and global cortical atrophy in participants aged < 75 years and ≥75 years.

Inflammatory biomarkers	<75 years group		≥75 years group	
	OR	*p*	OR	*p*
WBC	1.241 (0.828, 1.898)	0.304	0.775 (0.509, 1.176)	0.228
NEU	1.344 (0.898, 2.090)	0.166	0.709 (0.461, 1.085)	0.110
LYM	0.834 (0.545, 1.268)	0.395	1.000 (0.675, 1.511)	0.998
PLT	1.117 (0.734, 1.727)	0.609	0.790 (0.527, 1.188)	0.249
SII	1.531 (0.936, 2.623)	0.102	0.938 (0.668, 1.375)	0.715
SIRI	3.485 (1.222, 12.073)	0.036*	1.025 (0.692, 1.875)	0.917
NLR	1.587 (0.927, 2.870)	0.106	0.854 (0.588, 1.261)	0.402
MLR	2.701 (1.225, 6.708)	0.022*	1.241 (0.804, 2.176)	0.391
NPR	1.337 (0.793, 2.341)	0.288	0.721 (0.485, 1.066)	0.096
NMLR	1.686 (0.962, 3.139)	0.081	0.876 (0.603, 1.305)	0.486
AISI	2.515 (1.128, 6.534)	0.041*	1.035 (0.728, 1.806)	0.874

*p < 0.05.

### 3.5 Modifying effects of age and sex on the associations between inflammatory biomarkers and hippocampal atrophy

In the examination of the interaction effects between age and inflammatory biomarkers on hippocampal atrophy, the interaction terms for the NLR (OR = 0.935, *p* = 0.014), NPR (OR = 0.954, *p* = 0.042), and NMLR (OR = 0.939, *p* = 0.018) with age were found to be statistically significant. In contrast, the interaction terms for NEU (OR = 0.961, *p* = 0.066) and the SII (OR = 0.951, *p* = 0.071) demonstrated borderline significance. The interaction terms involving WBC, LYM, PLT, SIRI, MLR, and AISI with age did not achieve statistical significance. Regarding the interaction effects between sex and inflammatory biomarkers on hippocampal atrophy, only the interaction term between LYM and sex was statistically significant (OR = 2.479, *p* = 0.021). The interaction term between PLT and sex approached statistical significance (OR = 2.203, *p* = 0.074). The interaction terms between the remaining inflammatory biomarkers and sex were not statistically significant (Table 7).

**Table 7:** Interaction analyses of age, sex, and inflammatory biomarkers on hippocampal atrophy.

Inflammatory biomarkers	Age		Sex	
	OR	*p*	OR	*p*
WBC	0.981 (0.943, 1.020)	0.332	1.709 (0.837, 3.489)	0.141
NEU	0.961 (0.920, 1.003)	0.066	1.216 (0.604, 2.450)	0.584
LYM	1.027 (0.984, 1.071)	0.221	2.479 (1.148, 5.350)	0.021*
PLT	1.032 (0.984, 1.083)	0.194	2.203 (0.927, 5.231)	0.074
SII	0.951 (0.901, 1.004)	0.071	0.974 (0.455, 2.087)	0.946
SIRI	0.966 (0.909, 1.027)	0.266	0.636 (0.211, 1.917)	0.422
NLR	0.935 (0.886, 0.986)	0.014*	0.576 (0.280, 1.188)	0.135
MLR	0.994 (0.948, 1.042)	0.795	0.564 (0.243, 1.309)	0.182
NPR	0.954 (0.912, 0.998)	0.042*	0.748 (0.390, 1.434)	0.382
NMLR	0.939 (0.891, 0.989)	0.018*	0.570 (0.275, 1.183)	0.131
AISI	0.975 (0.919, 1.035)	0.410	1.032 (0.351, 3.033)	0.954

*p < 0.05.

Following the results from the aforementioned interaction analyses, age-stratified analyses were conducted to more clearly elucidate the modifying effects. Distinct multivariable logistic regression models were developed for individuals aged < 75 years and those aged ≥ 75 years. Within the < 75 years cohort, the SII (OR = 1.771, *p* = 0.049), NLR (OR = 2.304, *p* = 0.016), and NMLR (OR = 2.290, *p* = 0.019) exhibited significant associations with hippocampal atrophy. Conversely, other inflammatory biomarkers did not demonstrate significant associations with hippocampal atrophy. In the cohort aged ≥ 75 years, none of the inflammatory biomarkers were significantly associated with hippocampal atrophy (Table 8).

**Table 8:** Associations between inflammatory biomarkers and hippocampal atrophy in participants aged < 75 years and ≥75 years.

Inflammatory biomarkers	<75 years group		≥75 years group	
	OR	*p*	OR	*p*
WBC	1.105 (0.729, 1.668)	0.630	0.920 (0.617, 1.355)	0.675
NEU	1.339 (0.874, 2.113)	0.187	0.905 (0.585, 1.370)	0.643
LYM	0.703 (0.426, 1.118)	0.148	0.890 (0.601, 1.300)	0.550
PLT	0.900 (0.539, 1.481)	0.681	1.011 (0.682, 1.492)	0.958
SII	1.771 (1.018, 3.225)	0.049*	1.220 (0.812, 1.822)	0.324
SIRI	1.738 (0.802, 4.443)	0.188	1.276 (0.914, 1.932)	0.157
NLR	2.304 (1.215, 4.807)	0.016*	1.107 (0.736, 1.638)	0.613
MLR	1.189 (0.637, 2.301)	0.582	1.323 (0.918, 1.952)	0.133
NPR	1.418 (0.803, 2.595)	0.233	0.850 (0.586, 1.196)	0.363
NMLR	2.290 (1.194, 4.818)	0.019*	1.137 (0.760, 1.677)	0.518
AISI	1.520 (0.772, 3.221)	0.236	1.214 (0.895, 1.723)	0.202

*p < 0.05.

## 4 Discussion

This study conducted a systematic evaluation of the relationships between peripheral blood inflammatory biomarkers and cognitive function in patients with AD and AD-MCI, utilizing cross-sectional data. The analysis did not reveal significant associations between inflammatory biomarkers and cognitive function across the entire sample. However, interaction and stratified analyses indicated that inflammatory biomarkers were associated with cognitive function and brain structural abnormalities in individuals younger than 75 years. In contrast, no significant associations were found in participants aged 75 years and older. These findings suggest that age may modify the associations of peripheral inflammatory status with cognitive performance and structural brain abnormalities. Among the inflammatory biomarkers assessed, composite inflammatory indices demonstrated stronger associations and may serve as potential biomarkers for AD.

Chronic peripheral inflammation is recognized as a significant factor in the pathogenesis of various diseases (35). The relationship between chronic peripheral inflammation and AD has garnered substantial attention in previous research (36–39). This type of inflammation is primarily characterized by increased levels of specific inflammatory cytokines or immune cells in the bloodstream, while the BBB meticulously regulates the entry of molecules and cells from the circulatory system into the central nervous system (CNS). Consequently, BBB disruption may serve as a critical mechanism that connects peripheral inflammation to the CNS (15). Elevated levels of inflammatory cytokines and immune cells in the blood can compromise normal BBB function through several potential mechanisms, such as the inhibition of tight junctions in endothelial cells (14) and the stimulation of cerebral vascular endothelial cells and pericytes to produce prostaglandins (PG) and nitric oxide (NO) (40). Furthermore, BBB dysfunction itself constitutes a significant aspect of AD pathophysiology (41). Throughout the development and progression of AD, various components of the BBB, including endothelial cells, the basement membrane, pericytes, and astrocytes, exhibit abnormalities that disrupt normal BBB function (42, 43). This disruption allows inflammatory cytokines and immune cells from the bloodstream to infiltrate the CNS, where they activate microglia and astrocytes to produce inflammatory mediators, further exacerbating BBB disruption and advancing neurodegeneration.

The MMSE and structural neuroimaging provided complementary information in the present study: MMSE reflected global cognitive performance, whereas GCA and MTA scores reflected structural brain abnormalities. This study found that the associations of peripheral inflammatory biomarkers with MMSE differed across age groups within patients with AD and AD-MCI, highlighting the heterogeneity of these biomarker–outcome relationships in older adults. Numerous studies have corroborated the influence of age on chronic inflammation, generally observing an increase in inflammatory biomarker levels with advancing age (44–46). Consistent with these observations, participants aged ≥75 years had higher levels of inflammatory biomarkers than those aged <75 years (Supplementary Table S5). When inflammatory levels are uniformly elevated among most participants, their capacity to explain variations in cognitive function may be diminished. Furthermore, relatively younger individuals typically possess greater neuroplasticity and cognitive reserve (47), which may render them more susceptible to the effects of chronic inflammation on cognitive function. In contrast, cognitive decline in very old adults may be more significantly influenced by structural brain injury. Given that substantial cognitive impairment is often already present in this demographic, there may be limited potential for further decline. Consequently, the relationship between chronic inflammation and cognitive function scores may be attenuated in the ≥75 years age group, making it more challenging to detect, particularly in studies with limited sample sizes.

The analyses of the associations between inflammatory biomarkers and brain structural abnormalities revealed a similar pattern. In the cohort aged less than 75 years, significant associations were identified between SIRI, MLR, and AISI with global cortical atrophy, and between SII, NLR, and NMLR with hippocampal atrophy. These findings suggest that peripheral inflammatory status may remain more closely associated with brain structural abnormalities in patients aged <75 years. Conversely, in the cohort aged 75 years and above, no significant associations were detected between inflammatory biomarkers and brain structural abnormalities. This lack of association may be attributed to several factors. As individuals age further, the progression of brain atrophy is influenced by a multitude of factors, including neurodegenerative pathological burden, cerebral small vessel disease, nutritional status, and frailty (48–50). In such contexts, the independent contribution of inflammation to brain structural changes may be partially obscured by these more dominant factors. Second, older adults typically experience a phenomenon known as inflammaging, characterized by a generally elevated level of low-grade chronic inflammation (22). This condition may diminish the discriminatory capacity of inflammatory biomarkers among individuals, thereby weakening their associations with structural abnormalities in the brain. Moreover, structural abnormalities in the brain are more prevalent in individuals aged 75 years and older (51, 52), potentially further attenuating the relationship between peripheral inflammatory biomarkers and brain atrophy. These explanations align with those proposed above regarding the age-dependent associations between inflammatory biomarkers and cognitive performance.

Furthermore, the findings indicated a certain degree of specificity in the associations between various inflammatory biomarkers and distinct types of brain structural abnormalities. Global cortical atrophy often reflects more extensive neurodegenerative changes, the cumulative impact of cerebral small vessel disease, and age-related brain tissue loss at a systemic level (48). The biomarkers identified in this study – SIRI, MLR, and AISI – are all related to monocyte activity, suggesting a potential link between global cortical atrophy and peripheral inflammation involving monocytes. However, this proposed mechanism warrants further investigation. The hippocampus, a critical structure for learning and memory, is notably susceptible to inflammatory responses (53). Previous research has indicated that factors such as neutrophil activation, immune-inflammatory imbalance, and the escalation of neuroinflammation may contribute to neuronal damage in the hippocampus and impair synaptic plasticity (54, 55). Consequently, the associations of NLR, NMLR, and SII with hippocampal atrophy possess a degree of biological plausibility. However, hippocampal atrophy was defined using age-specific MTA cutoffs. Although age was adjusted for in the whole-sample regression models, the outcome definition itself varied by age, which may complicate the interpretation of biomarker–MTA associations in the overall cohort.

In comparison to age, the influence of sex as a modifying factor appears to be less pronounced. Although earlier studies have suggested that variations in sex hormone levels and metabolic backgrounds might affect the relationship between inflammatory status and cognitive decline or brain atrophy, no significant sex interaction was detected in the current study. The study population was characterized by an advanced age demographic, which may have diminished the impact of hormonal differences between postmenopausal women and men compared to earlier life stages. On the other hand, the influence of sex on brain structural abnormalities might manifest more prominently as a long-term cumulative effect rather than as an immediate modifier of the relationship between current peripheral inflammatory biomarkers and brain atrophy. Furthermore, the study’s sample size may have been inadequate. These factors could account for the absence of a significant sex-related modifying effect.

Overall, the study’s findings suggest a complex relationship between chronic peripheral inflammation and AD, potentially modulated by factors such as age. A notably innovative aspect of this study was the systematic analysis of multiple inflammatory biomarkers. The results indicated that composite inflammatory indices exhibited a stronger association with cognitive function than individual biomarkers, suggesting their potential utility as biomarkers for AD. This study is subject to several limitations. Firstly, it was conducted as a single-center investigation with a relatively small sample size, which was further diminished following age stratification. This reduction in sample size resulted in limited statistical power, potentially contributing to the absence of statistical significance for certain inflammatory biomarkers. Secondly, the study did not include an age-matched cognitively normal control group. Therefore, although the absence of healthy controls does not preclude the evaluation of biomarker–outcome associations within patients with AD and AD-MCI, it limits our ability to determine whether the observed inflammatory profiles are specific to these conditions or reflect changes associated with normal aging. Thirdly, no formal correction for multiple comparisons was applied; therefore, the nominally significant findings should be interpreted cautiously and require independent validation. Lastly, the cross-sectional design of this study precludes the determination of temporal relationships between inflammatory biomarkers and cognitive function. Future prospective studies are warranted to elucidate the causal relationship between these variables.

## 5 Conclusion

In patients with AD and AD-MCI, the associations of several peripheral inflammatory biomarkers with cognitive performance and brain structural abnormalities differed according to age and were more evident in patients aged <75 years. Compared with peripheral blood inflammatory biomarkers, several composite inflammatory indices showed better associations with cognitive scores and brain structural abnormalities. However, their prognostic, disease-specific, and diagnostic value requires validation in prospective studies.

## Data Availability

The raw data supporting the conclusions of this article will be made available by the authors, without undue reservation.
