# Supplementary material for: Age-dependent associations of peripheral inflammatory biomarkers with cognition and brain structural abnormalities in patients with Alzheimer’s disease and Alzheimer’s disease-related mild cognitive impairment
Source: Front Neurol. 2026 Sep 8;17:1868357. doi: 10.3389/fneur.2026.1868357 (PMC13593470; doi:10.3389/fneur.2026.1868357)
Supplement: Supplementary file 1 [file Data_Sheet_1.PDF]

Table S1. Associations of inflammatory biomarkers with cognitive score, global cortical atrophy, and hippocampal atrophy

Inflammatory biomarkers	model 1		model 2		model 3	
	β	*p*	β	*p*	β	*p*
WBC	-0.261	0.448	-0.152	0.633	-0.459	0.338
NEU	-0.489	0.155	-0.416	0.192	-0.655	0.440
LYM	0.368	0.283	0.444	0.168	0.343	0.394
PLT	-0.045	0.896	-0.35	0.301	-0.251	0.840
SII	-0.519	0.134	-0.559	0.081	-0.46	0.045*
SIRI	-0.392	0.260	-0.37	0.261	-0.456	0.227
NLR	-0.541	0.115	-0.467	0.147	-0.473	0.385
MLR	-0.243	0.494	-0.163	0.638	-0.297	0.207
NPR	-0.408	0.236	-0.202	0.544	-0.449	0.846
NMLR	-0.528	0.124	-0.454	0.161	-0.472	0.179
AISI	-0.467	0.200	-0.506	0.138	-0.541	0.414

* $p < 0.05$.

Table S2. Associations of inflammatory biomarkers with global cortical atrophy

Inflammatory biomarkers	model 1		model 2		model 3	
	OR	*p*	OR	*p*	OR	*p*
WBC	1.172	0.231	1.032	0.825	0.903	0.562
NEU	1.201	0.185	1.034	0.812	0.878	0.452
LYM	0.926	0.523	0.939	0.637	0.933	0.697
PLT	0.865	0.248	0.983	0.904	1.025	0.892
SII	1.221	0.170	1.164	0.306	1.087	0.651
SIRI	2.050	0.010*	1.469	0.155	1.268	0.418
NLR	1.259	0.116	1.089	0.567	0.980	0.907
MLR	1.886	0.002*	1.607	0.032*	1.701	0.057
NPR	1.187	0.221	0.898	0.479	0.779	0.135
NMLR	1.309	0.075	1.121	0.454	1.010	0.956
AISI	1.762	0.025*	1.472	0.114	1.353	0.300

* $p < 0.05$.

Table S3. Associations of inflammatory biomarkers with hippocampal atrophy

Inflammatory biomarkers	model 1		model 2		model 3	
	OR	*p*	OR	*p*	OR	*p*
WBC	1.054	0.691	1.015	0.915	1.074	0.684
NEU	1.119	0.415	1.062	0.675	1.028	0.876
LYM	0.864	0.275	0.893	0.411	0.999	0.995
PLT	0.856	0.251	1.003	0.984	1.057	0.790
SII	1.214	0.183	1.247	0.161	1.146	0.491
SIRI	1.309	0.140	1.182	0.330	1.231	0.291
NLR	1.283	0.084	1.181	0.271	1.019	0.914
MLR	1.256	0.103	1.146	0.361	1.188	0.338
NPR	1.101	0.454	0.937	0.645	0.905	0.549
NMLR	1.295	0.076	1.186	0.263	1.037	0.836
AISI	1.172	0.251	1.141	0.373	1.310	0.246

* $p < 0.05$.

Table S4. Baseline Characteristics Stratified by Age

Baseline Characteristics	< 75 years (n = 189)	≥ 75 years (n = 206)	*p*
Age (years)	70 (63, 72)	82 (79, 86)	
Sex			0.708
Female	106 (55.9)	120 (58.3)	
Male	83 (44.1)	86 (41.7)	
Education†			0.068
Primary school	25 (14.2)	31 (16.1)	
Middle school	117 (66.5)	105 (54.7)	
College or above	34 (19.3)	56 (29.2)	
Marital status†			0.019*
Married	159 (87.4)	153 (77.7)	
Other	23 (12.6)	44 (22.3)	
Smoking	14 (12.0)	11 (8.9)	0.565
Alcohol	14 (12.0)	7 (5.6)	0.131

Hypertension	82 (45.8)	96 (50.0)	0.482
Diabetes mellitus	60 (33.5)	62 (32.3)	0.888
CHD	14 (7.8)	28 (14.6)	0.059
Stroke	11 (6.1)	18 (9.4)	0.335
BMI (kg/m^2)	23.57±2.96	23.36±3.45	0.624
WBC (10^9/L)	210.85±54.82	189.53±55.12	<0.001*
LYM (10^9/L)	5.70 (4.70, 7.02)	5.84 (4.96, 7.19)	0.365
NEU (10^9/L)	1.82±0.59	1.68±0.66	0.035*
PLT (10^9/L)	3.54±1.35	3.77±1.36	0.100
SII	385 (263, 578)	400 (277, 600)	0.377
SIRI	0.73 (0.48, 1.03)	0.92 (0.58, 1.39)	<0.001*
NLR	1.88 (1.32, 2.69)	2.20 (1.57, 3.09)	0.002*
MLR	0.21 (0.17, 0.27)	0.25 (0.19, 0.35)	<0.001*
NPR	0.02 (0.01, 0.02)	0.02 (0.02, 0.03)	<0.001*
NMLR	2.13 (1.50, 2.93)	2.50 (1.78, 3.39)	0.002*
AISI	145 (95, 231)	160 (100, 274)	0.092

* $p < 0.05$.

†There were 27 missing values for educational level and 16 missing values for marital status, while no missing values were observed for the other variables.
