# Supplementary material for: Age-dependent associations of peripheral inflammatory biomarkers with cognition and brain structural abnormalities in patients with Alzheimer’s disease and Alzheimer’s disease-related mild cognitive impairment
Source: Front Neurol. 2026 Sep 8;17:1868357. doi: 10.3389/fneur.2026.1868357 (PMC13593470; doi:10.3389/fneur.2026.1868357)

Table S1. Baseline characteristics of the participants

Baseline Characteristics	Overall (n = 395)	AD-MCI (n = 135)	AD (n = 260)	p
Age (years)	76 (70, 83)	71 (66, 77)	78 (72, 84)	<0.001*
Sex				0.871
Female	226 (57.1)	78 (57.8)	148 (56.9)	
Male	169 (42.9)	57 (42.2)	112 (43.1)	
Education†				0.835
Primary school	56 (15.2)	15 (13.5)	41 (16.0)	
Middle school	222 (60.3)	68 (61.3)	154 (59.9)	
College or above	90 (24.5)	28 (25.2)	62 (24.2)	
Marital status†				0.605
Married	310 (81.8)	109 (83.2)	201 (81.0)	
Other	69 (18.2)	22 (16.8)	47 (19.0)	
Smoking	41 (10.4)	19 (14.1)	22 (8.5)	0.083
Alcohol	34 (8.7)	14 (10.4)	20 (7.7)	0.368
Hypertension	182 (46.1)	58 (43.0)	124 (47.7)	0.371
Diabetes mellitus	127 (32.2)	38 (28.1)	89 (34.2)	0.220
CHD	44 (11.1)	10 (7.4)	34 (13.1)	0.090
Stroke	30 (7.6)	6 (4.4)	24 (9.2)	0.089
BMI (kg/m^2)	23.46 ± 3.22	24.19 ± 3.02	23.22 ± 3.26	0.060
WBC (10^9/L)	5.79 (4.80, 7.11)	5.68 (4.80, 6.70)	5.82 (4.82, 7.20)	0.292
LYM (10^9/L)	1.75 ± 0.63	1.83 ± 0.68	1.72 ± 0.61	0.122
NEU (10^9/L)	3.66 ± 1.36	3.47 ± 1.14	3.74 ± 1.44	0.078
PLT (10^9/L)	199.81 ± 55.93	202.05 ± 58.18	198.86 ± 55.02	0.616
SII	397 (272, 587)	357 (282, 505)	418 (269, 622)	0.082
SIRI	0.79 (0.51, 1.21)	0.70 (0.47, 1.00)	0.89 (0.54, 1.33)	0.002*
NLR	2.05 (1.47, 2.81)	1.82 (1.38, 2.42)	2.18 (1.51, 3.00)	0.017*
MLR	0.23 (0.18, 0.31)	0.21 (0.17, 0.28)	0.24 (0.19, 0.33)	0.004*
NPR	0.02 (0.01, 0.02)	0.02 (0.01, 0.02)	0.02 (0.01, 0.02)	0.079

NMLR	2.31 (1.66, 3.11)	2.03 (1.54, 2.69)	2.45 (1.71, 3.33)	0.009*
AISI	155 (96, 256)	135 (94, 198)	159 (98, 272)	0.011*
MMSE	17 (12, 21)	20 (17, 23)	15 (10, 19)	<0.001*

*p < 0.05.

†There were 27 missing values for educational level and 16 missing values for marital status, while no missing values were observed for the other variables.

Table S2. Associations of inflammatory biomarkers with cognitive score, global cortical atrophy, and hippocampal atrophy

Inflammatory biomarkers	model 1		model 2		model 3	
	β	*p*	β	*p*	β	*p*
WBC	-0.261	0.448	-0.152	0.633	-0.459	0.338
NEU	-0.489	0.155	-0.416	0.192	-0.655	0.440
LYM	0.368	0.283	0.444	0.168	0.343	0.394
PLT	-0.045	0.896	-0.35	0.301	-0.251	0.840
SII	-0.519	0.134	-0.559	0.081	-0.46	0.045*
SIRI	-0.392	0.260	-0.37	0.261	-0.456	0.227
NLR	-0.541	0.115	-0.467	0.147	-0.473	0.385
MLR	-0.243	0.494	-0.163	0.638	-0.297	0.207
NPR	-0.408	0.236	-0.202	0.544	-0.449	0.846
NMLR	-0.528	0.124	-0.454	0.161	-0.472	0.179
AISI	-0.467	0.200	-0.506	0.138	-0.541	0.414

* $p < 0.05$.

Table S3. Associations of inflammatory biomarkers with global cortical atrophy

Inflammatory biomarkers	model 1		model 2		model 3	
	OR	*p*	OR	*p*	OR	*p*
WBC	1.172	0.231	1.032	0.825	0.903	0.562
NEU	1.201	0.185	1.034	0.812	0.878	0.452
LYM	0.926	0.523	0.939	0.637	0.933	0.697
PLT	0.865	0.248	0.983	0.904	1.025	0.892

SII	1.221	0.170	1.164	0.306	1.087	0.651
SIRI	2.050	0.010*	1.469	0.155	1.268	0.418
NLR	1.259	0.116	1.089	0.567	0.980	0.907
MLR	1.886	0.002*	1.607	0.032*	1.701	0.057
NPR	1.187	0.221	0.898	0.479	0.779	0.135
NMLR	1.309	0.075	1.121	0.454	1.010	0.956
AISI	1.762	0.025*	1.472	0.114	1.353	0.300

* $p < 0.05$.

Table S4. Associations of inflammatory biomarkers with hippocampal atrophy

Inflammatory biomarkers	model 1		model 2		model 3	
	OR	*p*	OR	*p*	OR	*p*
WBC	1.054	0.691	1.015	0.915	1.074	0.684
NEU	1.119	0.415	1.062	0.675	1.028	0.876
LYM	0.864	0.275	0.893	0.411	0.999	0.995
PLT	0.856	0.251	1.003	0.984	1.057	0.790
SII	1.214	0.183	1.247	0.161	1.146	0.491
SIRI	1.309	0.140	1.182	0.330	1.231	0.291
NLR	1.283	0.084	1.181	0.271	1.019	0.914
MLR	1.256	0.103	1.146	0.361	1.188	0.338
NPR	1.101	0.454	0.937	0.645	0.905	0.549
NMLR	1.295	0.076	1.186	0.263	1.037	0.836
AISI	1.172	0.251	1.141	0.373	1.310	0.246

* $p < 0.05$.

Table S5. Baseline Characteristics Stratified by Age

Baseline Characteristics	< 75 years (n = 189)	≥ 75 years (n = 206)	*p*
Age (years)	70 (63, 72)	82 (79, 86)	
Sex			0.708

Female	106 (55.9)	120 (58.3)	
Male	83 (44.1)	86 (41.7)	
Education†			0.068
Primary school	25 (14.2)	31 (16.1)	
Middle school	117 (66.5)	105 (54.7)	
College or above	34 (19.3)	56 (29.2)	
Marital status†			0.019*
Married	159 (87.4)	153 (77.7)	
Other	23 (12.6)	44 (22.3)	
Smoking	14 (12.0)	11 (8.9)	0.565
Alcohol	14 (12.0)	7 (5.6)	0.131
Hypertension	82 (45.8)	96 (50.0)	0.482
Diabetes mellitus	60 (33.5)	62 (32.3)	0.888
CHD	14 (7.8)	28 (14.6)	0.059
Stroke	11 (6.1)	18 (9.4)	0.335
BMI (kg/m^2)	23.57±2.96	23.36±3.45	0.624
WBC (10^9/L)	210.85±54.82	189.53±55.12	<0.001*
LYM (10^9/L)	5.70 (4.70, 7.02)	5.84 (4.96, 7.19)	0.365
NEU (10^9/L)	1.82±0.59	1.68±0.66	0.035*
PLT (10^9/L)	3.54±1.35	3.77±1.36	0.100
SII	385 (263, 578)	400 (277, 600)	0.377
SIRI	0.73 (0.48, 1.03)	0.92 (0.58, 1.39)	<0.001*
NLR	1.88 (1.32, 2.69)	2.20 (1.57, 3.09)	0.002*
MLR	0.21 (0.17, 0.27)	0.25 (0.19, 0.35)	<0.001*
NPR	0.02 (0.01, 0.02)	0.02 (0.02, 0.03)	<0.001*
NMLR	2.13 (1.50, 2.93)	2.50 (1.78, 3.39)	0.002*
AISI	145 (95, 231)	160 (100, 274)	0.092

* $p < 0.05$.

†There were 27 missing values for educational level and 16 missing values for marital status, while no missing values were observed for the other variables.

Figure S1. Age-stratified associations of NLR, NPR, and NMLR with predicted MMSE scores.

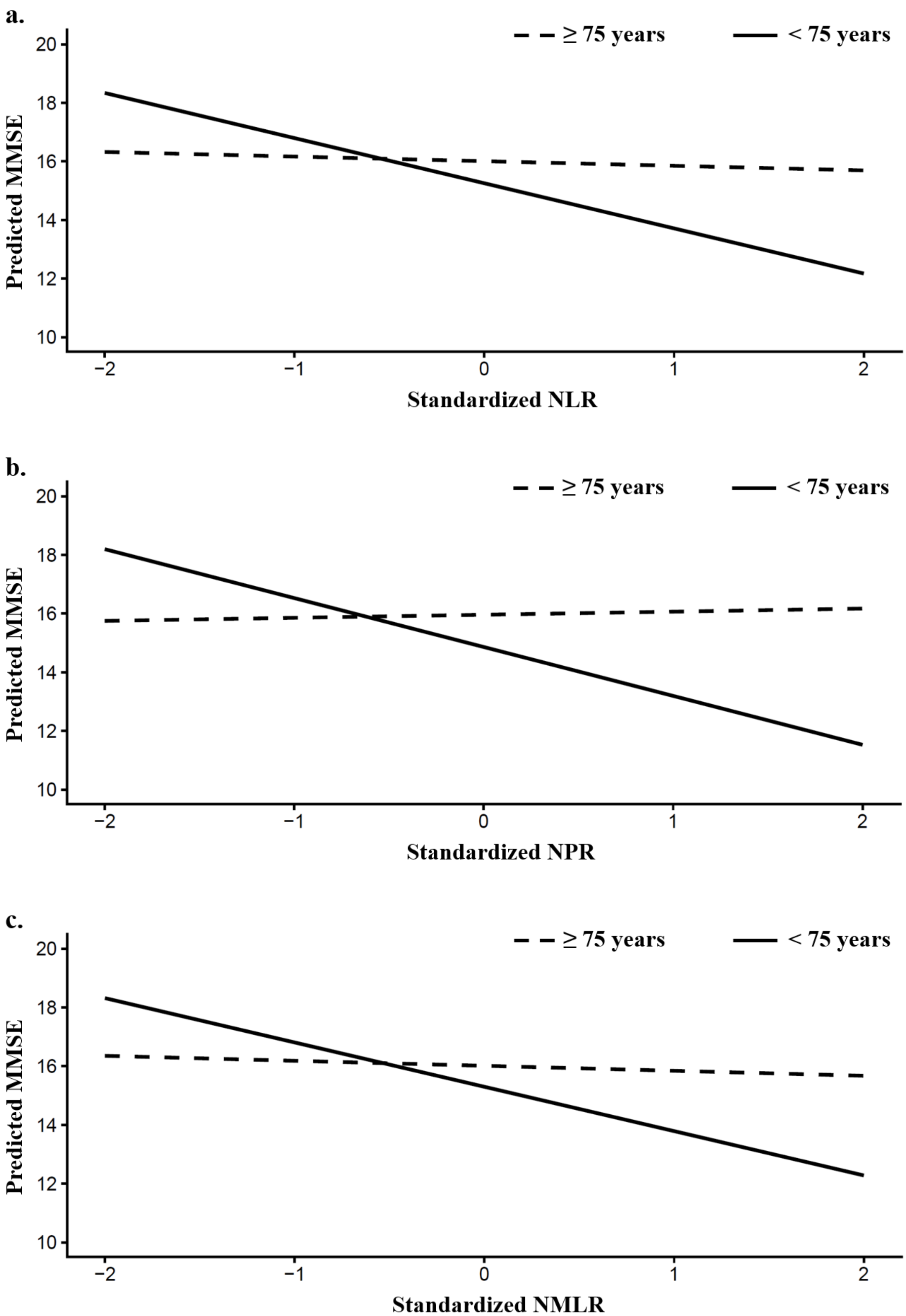

Figure S2. Age-stratified associations of SIRI, MLR, and AISI with predicted probability of global cortical atrophy.

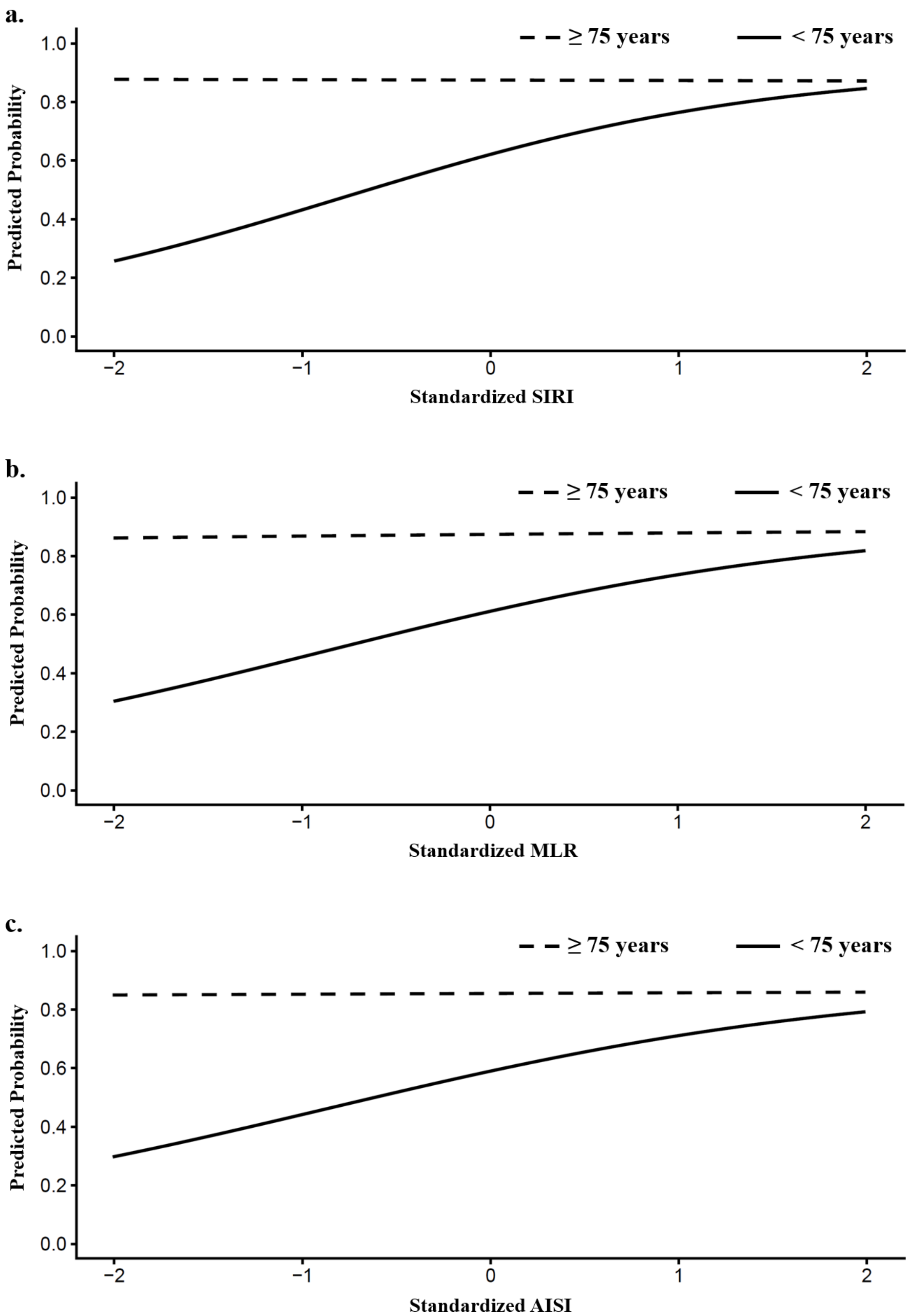

Figure S3. Age-stratified associations of SII, NLR, and NMLR with predicted probability of hippocampal atrophy.

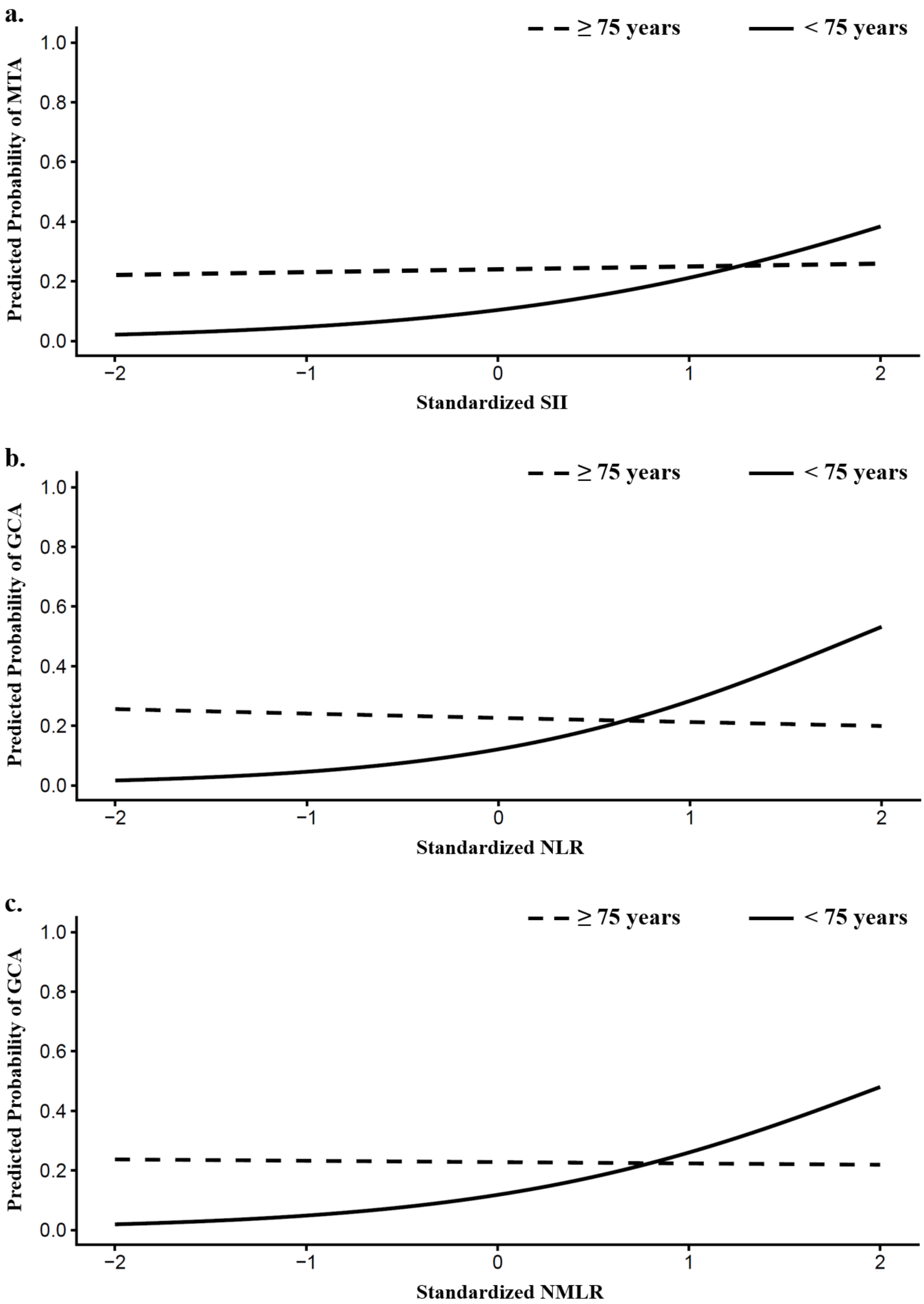

Supplement: Supplementary file 2 [file Data_Sheet_2.PDF]
